# Facile synthesis of iron oxides/reduced graphene oxide composites: application for electromagnetic wave absorption at high temperature

**DOI:** 10.1038/srep09298

**Published:** 2015-03-19

**Authors:** Lili Zhang, Xinxin Yu, Hongrui Hu, Yang Li, Mingzai Wu, Zhongzhu Wang, Guang Li, Zhaoqi Sun, Changle Chen

**Affiliations:** 1 School of Physics and Materials Science, Anhui University, Hefei 230601, China; 2 CAS Key Laboratory of Soft Matter Chemistry, Department of Polymer Science & Engineering, University of Science and Technology of China, Hefei 230026, China

## Abstract

Iron oxides/reduced graphene oxide composites were synthesized by facile thermochemical reactions of graphite oxide and FeSO_4_·7H_2_O. By adjusting reaction temperature, α-Fe_2_O_3_/reduced graphene oxide and Fe_3_O_4_/reduced graphene oxide composites can be obtained conveniently. Graphene oxide and reduced graphene oxide sheets were demonstrated to regulate the phase transition from α-Fe_2_O_3_ to Fe_3_O_4_ via γ-Fe_2_O_3_, which was reported for the first time. The hydroxyl groups attached on the graphene oxide sheets and H_2_ gas generated during the annealing of graphene oxide are believed to play an important role during these phase transformations. These samples showed good electromagnetic wave absorption performance due to their electromagnetic complementary effect. These samples possess much better electromagnetic wave absorption properties than the mixture of separately prepared Fe_3_O_4_ with rGO, suggesting the crucial role of synthetic method in determining the product properties. Also, these samples perform much better than commercial absorbers. Most importantly, the great stability of these composites is highly advantageous for applications as electromagnetic wave absorption materials at high temperatures.

Nowadays, severe electromagnetic (EM) radiation is being generated everywhere due to the increasing use of wireless communication tools, local area network, personal digital devices, and so on. EM radiation has become a serious pollution issue, not only influencing the operation of electronic devices, but also affecting human health and raising problems concerning their military applications^1^,^2^. In this regard, high performance EM wave absorption materials have attracted more and more attention as an effective strategy to solve these problems. The desired properties for ideal EM wave absorption materials include strong absorption capability, wide absorption range, lightweight, good thermal and oxidation stability, etc. Most conventional EM wave absorption materials are magnetic or metallic particles with electromagnetic parameters not functioning well in the GHz range. Combining with the high density and phase instability, their practical applications have been greatly limited. In contrast, carbon-based materials (carbon black, graphite flakes, carbon fiber, carbon nanotubes, reduced graphene oxide, etc) could potentially solve these issues due to their unique properties such as low density, high complex permittivity and superior thermal stability. Unfortunately, their EM wave absorption property mainly originates from dielectric loss because of their non-magnetic feature. The preparation of carbon-based composite materials with magnetic particles could efficiently solve this problem via controllable modifications of their dielectric and magnetic properties^3^,^4^.

Recently, rGO was reported to demonstrate enhanced EM wave absorption, comparing with graphite, carbon nanotubes and high quality graphene^5^. This was attributed to their defects and functional groups. However, the value of EM wave absorption is only −7 dB^5^, −3 dB away from the minimum requirement for practical applications (−10 dB). Various iron oxides/rGO composites have been explored to address this issue. Recently, Zhang et al. reported a maximum absorption of −33.5 dB from a rGO/α-Fe_2_O_3_ composite hydrogel prepared via a two-step process^3^. He et al. reported a facile solvothermal route to prepare laminated rGO/Fe_3_O_4_ composites, with reflection loss (RL) below −10 dB at 2 GHz and a maximum absorption of −26.4 dB^6^. Yin et al. fabricated rGO/γ-Fe_2_O_3_ composite with RL of −59.65 dB at 10.09 GHz^7^. Among these fabrication methods including hydrothermal, solvothermal, sol-gel process and chemical route^3^,^6^,^7^,^8^,^9^, most of them suffer from complicated and time-consuming procedures, which greatly limit their potential large scale application. The development of a facile, cost-effective and scalable method to synthesize iron oxides/rGO composite with high EM wave absorbing performance is highly desired.

A huge disadvantage of the conventional magnetic absorbing materials is the loss magnetic properties and consequently EM wave absorbing properties under high temperatures^10^. In fact, the temperature increment due to the conversion of electromagnetic energy into heat may cause serious damage to magnetic absorbing materials and related^11^. This is especially true for military stealth materials for radar cross section (RCS) reduction. The heat generated on the surface of hyper-velocity missiles, bombers, rockets, aircrafts and spacecrafts due to friction can result in high temperatures (600–800°C), leading to composition changes and even destruction the EM wave absorbing materials.

Herein, we report a simple, efficient and scalable procedure for the synthesis of iron oxides/rGO composite from thermochemical oxidation of FeSO_4_·7H_2_O and reduction of graphite oxide. Interestingly, the initially formed α-Fe_2_O_3_/rGO composite was converted to Fe_3_O_4_/rGO composite via γ-Fe_2_O_3_/rGO intermediate when the temperature was increased from 500°C to 800°C. In contrast, treating FeSO_4_·7H_2_O powders without the aid of rGO under the same conditions generated α-Fe_2_O_3_ at every temperature point. This is the first example of phase transition between α-Fe_2_O_3_, γ-Fe_2_O_3_ and Fe_3_O_4_ regulated by rGO. All three composites showed great EM wave absorption abilities. More importantly, the thermal stability of the Fe_3_O_4_/rGO composite at up to 800°C may open up a whole new field for high temperature application of carbon-based composite materials as EM wave absorption materials.

## Results

### Experimental Procedure and Samples Labeling

Experimental details can be found in **Methods** section. The starting material S90 was obtained by drying FeSO_4_·7H_2_O and GO at 90°C in air for 24 h. After heating, the products were grounded in an agate mortar, annealed at a certain temperature and labeled as SX, where X denotes the treating temperature. The control samples were prepared by treating FeSO_4_·7H_2_O powders (without GO) under the same conditions, and labeled as S_0_X, where X denotes the treating temperature.

### X-ray Diffraction (XRD) and Raman Analysis

**Figure 1:**
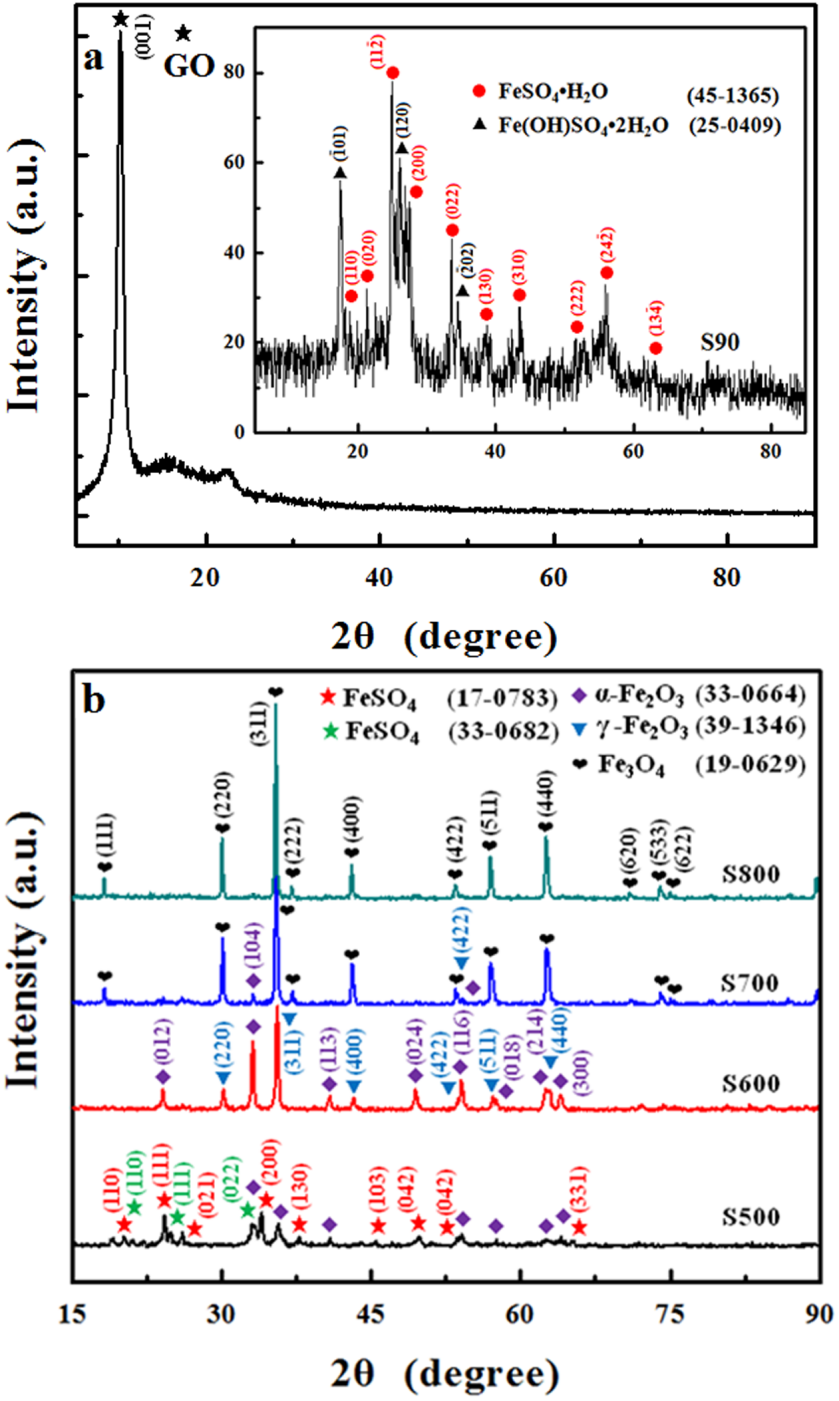
XRD patterns of (a) graphite oxide dried at 90°C and S90 (inset), (b) S500, S600, S700 and S800.

XRD patterns of GO and S90 are shown in Figure 1a. The characteristic peak at 2θ = 10.2° is indexed to the (001) plane of GO, indicating the oxidation of graphite to graphite oxide. The disappearance of the peak at 2θ = 10.2° in the XRD patterns of S90 (inset in Figure 1a) is due to the interruption of the GO layered structures with the intercalation of Fe^2+^ and SO_4_^2−^ ions. All the peaks for S90 are well indexed to szomolnokite (FeSO_4_·H_2_O, JCPDS 45-1365) and butlerite (Fe(OH)SO_4_·7H_2_O, JCPDS 25-0409), resulting from the loss of water molecules and the partial oxidation of FeSO_4_ during the drying process at 90°C^12^,^13^. XRD patterns of control samples are shown in Supplementary Figure S1 in Supplementary Information.

Figure 1b shows the XRD patterns of samples S500, S600, S700 and S800. For S500, the appearance of iron sulfate (FeSO_4_, JCPDS 17-0783 and JCPDS 33-0682) can be attributed to the loss of water from FeSO_4_·7H_2_O at high temperature^13^,^14^, and the thermal decomposition of FeSO_4_ led to the formation of hematite (α-Fe_2_O_3_, JCPDS 33-0664)^12^. At 600°C, parts of hematite are transformed into maghemite (γ-Fe_2_O_3_, JCPDS 39-1346). Based on TG/DTG analysis and the XRD pattern of sample S540 (α-Fe_2_O_3_, Supplementary Figure S1), a mixture of α-Fe_2_O_3_ and γ- Fe_2_O_3 _was observed at the temperatures between 540°C and 700°C. At 700°C, the product was dominated by magnetite (Fe_3_O_4_, JCPDS 19-0629) with a little bit of hematite. At 800°C, only magnetite phase was observed. Based on the above results, a clear phase transition sequence between the three iron oxides can be summarized:
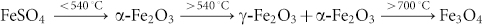


It is usually difficult to distinguish γ-Fe_2_O_3_ from Fe_3_O_4_ based on XRD analysis due to their analogous inverse spinel structures. Therefore, Raman analysis was carried out, which showed that sample S500 is mainly α-Fe_2_O_3_, S600 is the mixture of α-Fe_2_O_3_ and γ-Fe_2_O_3_, S700 and S800 are Fe_3_O_4_ (Supplementary Figure S2). These results are fully consistent with the XRD analysis.

### Transmission Electron Microcopy (TEM) Analysis

**Figure 2:**
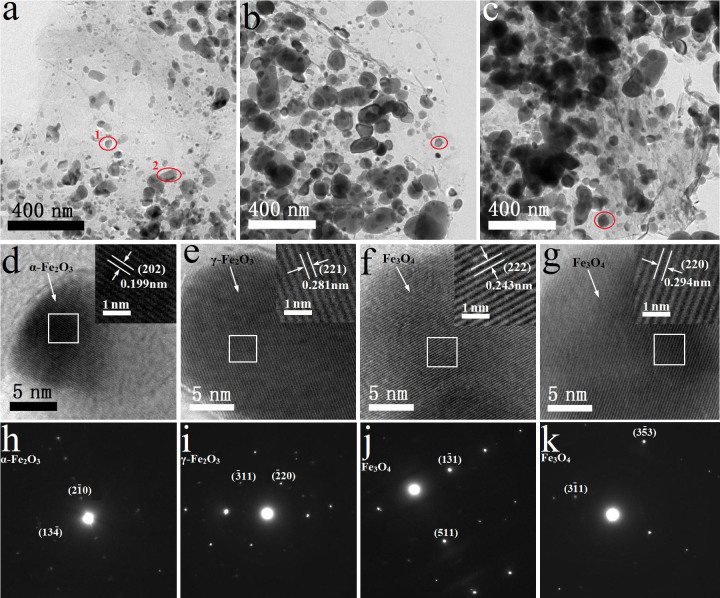
TEM images and SAED patterns. TEM images of (a) S600, (b) S700, (c) S800. HR-TEM images of (d, e) S600, (f) S700, (g) S800. SAED patterns of (h, i) S600, (j) S700, (k) S800. The red ellipses 1 and 2 in TEM image of “Figure 2a” correspond to SAED patterns of h and i, respectively. The red ellipse in TEM images of Figure 2b and Figure 2c correspond to SAED patterns of j and k, respectively.

Figure 2 shows the TEM images, HR-TEM images and SAED patterns of S600, S700 and S800. Many thin sheets of rGO decorated with iron oxides particles were detected, suggesting that they are easily exfoliated by sonication during the TEM test, since aggregation of rGO layers was observed in SEM images (Supplementary Figure S3). Iron oxide particles in the size of ~230 nm or smaller are well distributed on the surface of rGO sheets. HR-TEM images of d and e correspond to the (202) plane of α-Fe_2_O_3_ and (221) plane of γ-Fe_2_O_3_, and SAED patterns of h and i correspond to electron diffraction patterns of α-Fe_2_O_3_ and γ-Fe_2_O_3_. These results indicate that S600 is a mixture of α-Fe_2_O_3_ and γ-Fe_2_O_3_. The TEM, HR-TEM and SAED analysis of S700 and S800 (Figure 2 b, c, f, g, j and k) suggests the existence of only Fe_3_O_4_ phase. All of these results are consistent with XRD and Raman analysis. In addition, no significant morphological difference was observed among these three oxides.

### Fourier Transformed Infrared (FT-IR) and Thermogravimetric/Differential Thermogravimetry (TG/DTG) Analysis

In order to understand the mechanism of phase transitions of iron oxides, FT-IR and TG/DTG measurements were carried out and the results are shown in Supplementary Figure S4 and S5. The characteristic peaks of various carbon-oxygen functional groups from GO disappeared for S700 and S800, indicating the complete removal of epoxide and hydroxyl groups. For S800, only peaks of Fe_3_O_4_ are detected (Supplementary Figure S4). Compared with pure FeSO_4_·7H_2_O, the introduction of graphite oxide into S90 greatly reduces the starting decomposition temperature and the maximum weight loss temperature (510°C vs 430°C, 578°C vs 538°C). In addition, the endothermic peak at 538°C was identified as the decomposition temperature of FeSO_4_ to α-Fe_2_O_3_ (Supplementary Figure S5).

## Discussion

Generally, γ-Fe_2_O_3_ tends to convert to α-Fe_2_O_3_ under high temperatures^15^ or other extreme conditions^16^. Kachi pointed out that an orientation relationship (OR) exists when both phases coexist: (0001)_α_*∥*(111)_γ_ and [1–100]_α_*∥*[−110]_γ_^17^, which implies that a common plane with the hexagonal closed packing of oxygen ions remains undistorted after phase transformation. After decades of investigations, the conversion of α-Fe_2_O_3_ to γ-Fe_2_O_3_ can only be realized by mechanical grinding in ethanol^18^, high energy ball milling in ethanol^19^ and high temperature annealing of α-Fe_2_O_3_ in H_2_ atmosphere^20^,^21^. In the process of mechanical grinding and ball milling, ethanol is believed to play a key role in preventing the reduction of α-Fe_2_O_3_ and avoiding the formation of aggregates and favoring the rearrangement of O^2−^ from hexagonal closed packing to cubic closed packing. The exact mechanism behind the transformation of α-Fe_2_O_3_ to γ-Fe_2_O_3_ is still unclear.

Four main types of functional groups are believed to exist at the edges and on the surface of graphene oxide sheets: epoxide, carbonyl, carboxyl and hydroxyl^22^. Epoxide and hydroxyl groups are more stable than the other two kinds of functional groups^23^,^24^. This was confirmed in our FT-IR study, which showed that only epoxide and hydroxyl groups remained after annealing at 600°C. Based on the above analysis, a possible mechanism for the transformation from α-Fe_2_O_3_ to γ- Fe_2_O_3_ in our system is proposed. From 540°C to 700°C, the temperature field provides energy for the rearrangement of O^2−^ from hexagonal closed-packing to cubic closed-packing. The hydroxyl groups and H_2_ gas generated from the thermal reduction of graphite oxide are believed to play an important role in the transformation considering the similarities in conditions of our system with previously reported reaction systems. For example, the adsorbed hydroxyl groups have been reported to be crucial to the structural transformation from α-Fe_2_O_3_ into γ-Fe_2_O_3_, and the formation of γ-Fe_2_O_3_ phase started after the hydroxyl groups were exhausted^25^. Recently, ab initio calculations showed that the adsorption of H_2_ on the Fe-terminate α-Fe_2_O_3_ surface gained adsorption energy of −0.7 eV per H_2_ molecule and the angles and bond lengths of Fe-O bond were changed^21^. The chemical bonds in α-Fe_2_O_3_ have a tendency to those of γ-Fe_2_O_3_, implying that hydrogen plays a key factor to the phase transformation. Considering the complexity of our sample, the exact transformation mechanism is to be explored.

The transition from γ-Fe_2_O_3_ to Fe_3_O_4_ was believed to be facilitated by the porosity of the sample, which efficiently traps the reducing gases from the thermal reduction of graphite oxide. Nitrogen adsorption-desorption isotherm analysis was carried out for sample S800 (Supplementary Figure S6). The calculated specific surface area and the most probable pore diameter are 20.1 m^2^/g and 4 nm. It was shown before that the thermal reduction of graphite oxide could generate reducing gases including CO, CH_4_ and H_2_^26^,^27^. Because of its porous structure, sample S800 could adsorb and confine these reducing gases especially when the sample is dense solid, thus providing reducing atmosphere for the reduction of γ-Fe_2_O_3_ to Fe_3_O_4_.

In addition, this mechanism was supported by the fact that phase transition initially takes place in the inner part of samples with unchanged α-Fe_2_O_3_ shells covering the sample surface (Supplementary Figure S7 and Supplementary Figure S8). The reducing agents CH_4_, CO and H_2_ first reacted with the particles inner surface (Supplementary Figure S9a), removing the oxygen ions at the surface layers and transforming Fe^3+^ ions into Fe^2+^ ions. The diffusion of the initially formed Fe^2+^ ions from the surface into the lattice generates the corresponding positive charges. Therefore, electron hopping process took place from the ferrous ions at the surface to the ferric ions inside the lattice. Consequently, Fe^3+^ ions are regenerated at the surface and the reduction process continues. The concentration gradient difference of iron/oxygen ratio between the surface and the inner part of the particles is responsible for the diffusion process. Considering the dimensions of the particles, smaller grain size could facilitate the diffusion process. With the aid of rGO sheets, electron hoping between Fe^2+^ and Fe^3+^ ions in Fe_3_O_4_ may also take place between neighboring Fe_3_O_4_ particles^28^.

In support of the proposed mechanism, the grinding of S90 into powders is harmful to the α-Fe_2_O_3_ to Fe_3_O_4_ transition. As mentioned above, S800 contains only Fe_3_O_4_ phase. In contrast, annealing of S90 powders at 800°C afforded ground sample containing mainly α-Fe_2_O_3_ (nonmagnetic) phase as indicated by XRD analysis (Supplementary Figure S9b, S9c). M-H hysteresis measure at 300 K indicates that the saturated magnetization of the ground sample and S800 are 1.93 and 80.4 emu/g, respectively (Supplementary Figure S9d). The small magnetization of the ground sample suggests the presence of a small amount of magnetic iron oxides, which might be covered by the α-Fe_2_O_3_ phase and could not be detected by X-ray analysis due to limited penetration. The crumples of GO sheets (Supplementary Figure S3f) and loose accumulation of FeSO_4_·7H_2_O during the drying process are believed to confine the reducing gases and retard their effusion, facilitating the phase formation of α-Fe_2_O_3_.

As was shown above, the introduction of rGO sheets is beneficial to the stability of Fe_3_O_4_ phase at high temperature (800°C), which is highly desired for radar absorbing materials, especially for hyper-velocity missiles and spacecrafts. Therefore, the microwave absorption properties of sample S600, S700 and S800 were studied. The RL values were calculated using the relative complex permittivity and permeability at a given frequency and layer thickness based on the following equation (transmit line theory)^29^, 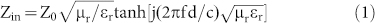

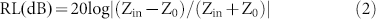
where *f* is the microwave frequency, *d* is the thickness of the absorber, *c* is the velocity of light, *Z*_0_ is the impedance of air, and *Z*_in_ is the input impedance of the absorber.

**Figure 3:**
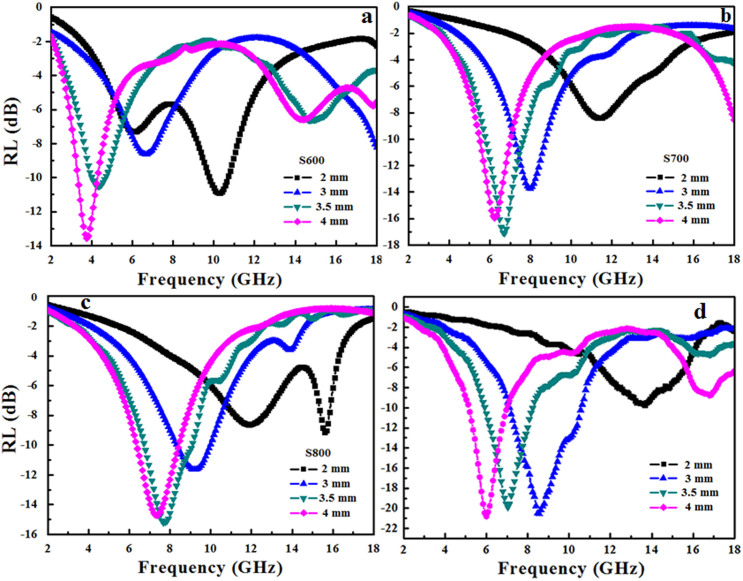
Electromagnetic RL of (a) S600, (b) S700, (c) S800, (d) sample of 10% GO in starting material treated at 700°C/paraffin wax composites versus frequency.

Figure 3 shows the relationship between the RL and frequency for sample S600, S700, S800 and the sample with 10% GO annealed at 700°C at different thickness. The minimum RL were found moving toward the lower frequency region with increasing thickness. Sample S600 with 4 mm in thickness shows the strongest absorption peak at 3.76 GHz with a minimum RL of −13.6 dB and a broad peak at 14.4 GHz with a minimum RL of −6.6 dB. Sample S700 and S800 with 3.5 mm in thickness have strong peaks at 6.72 GHz and 7.68 GHz with the minimum RL of −17.1 dB and −15.2 dB, respectively. The bandwidths of RL values below −10 dB are 0.96 GHz, 1.84 GHz and 2.35 GHz for S600, S700 and S800, respectively. Clearly, 3.5 mm is the optimal microwave absorption thickness for S700 and S800, which can be explained by the relationship between matching frequency and thickness^30^.
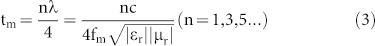


Where λ, μ_r_, and ε_r_ are the wavelength in the materials, complex permeability at f_m_, and complex permittivity at f_m_, f_m_ and t_m_ are the peak frequency and the matching thickness of maximum microwave absorptions, and c is the velocity of light. According to the eq. 3, the improved μ_r_ and ε_r_ of the materials are necessary to obtain small matching thickness t_m_ in lower frequency. These results are of importance since the absorption frequency ranges can be tuned easily by changing the thickness of the absorbers, and broadband absorption can be achieved by multilayered absorbing structure^31^.

When the GO dosage in the starting materials is increased from 5% to 10%, the microwave absorption performance of the sample annealed at 700°C is further improved, with RL value of −20.8 d B at 6 GHz (Figure 3d).

**Figure 4:**
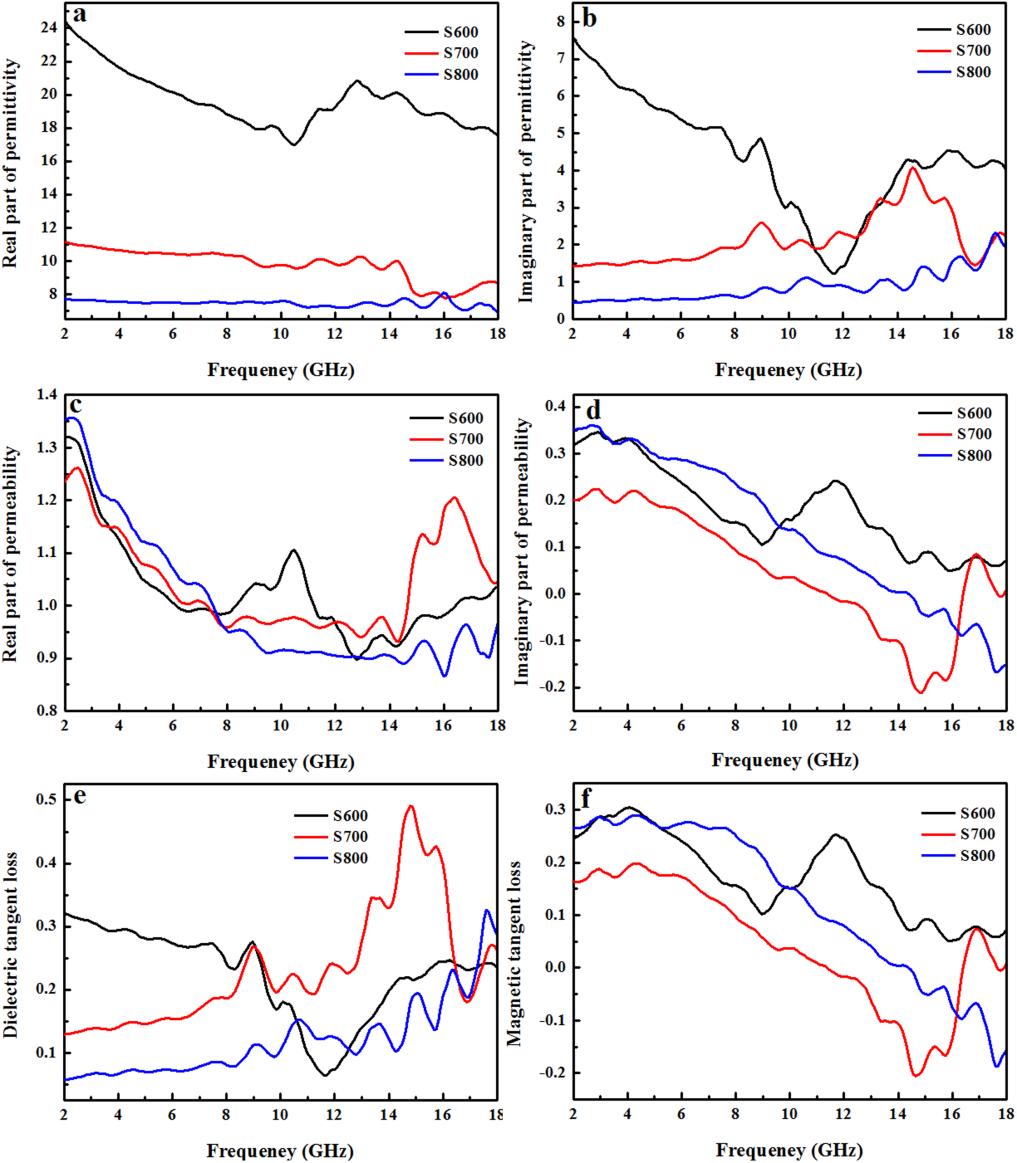
Frequency dependence on complex permittivity, complex permeability, dielectric tangent loss and magnetic tangent loss. Frequency dependence on (a) real part of complex permittivity, (b) imaginary part of complex permittivity, (c) real part of complex permeability, (d) imaginary part of complex permeability, (e) dielectric tangent loss, (f) magnetic tangent loss of S600, S700 and S800 with a thickness of 3 mm.

The microwave absorption properties of materials are related with the impedance matching between dielectric loss and magnetic loss. The imaginary parts of complex permittivity and complex permeability symbolize the loss of electrical and magnetic energy, respectively. Loss tangents (tanδ_E_ and tanδ_M_) represent the loss properties of incident electromagnetic wave in the microwave absorbent^32^. Higher values of ε″, μ″, tanδ_E_ and tanδ_M_ imply better performance of microwave absorption^6^,^33^. To investigate the possible mechanism of microwave absorption of the above three samples, the complex permittivity, complex permeability and corresponding dielectric and magnetic tangent loss of the samples with the thickness of 3 mm were measured at room temperature and the results are shown in Figure 4 and Table 1.

Figure 4a and 4b display the frequency dependence of the real part (ε′) and imaginary part (ε″) of the complex permittivity, which represent the energy storage and inner dissipation abilities, respectively. It can be seen that the ε′ of S600 first decreases from 24 to 17 in the range of 2–12 GHz, then increases and fluctuates and finally approaches a constant. On the other hand, the ε′ of S700 and S800 are about 8 and 11 at 2 GHz, respectively and stay constant up to 8 GHz and show a small fluctuation between 8 and 18 GHz, indicating a resonance behavior. The ε″ of S600 decrease first and then increase to 4.8 with a minor fluctuation in the 11.7–18 GHz range. For S700 and S800, the ε″ are about 0.5 and 1.5 at 2 GHz, respectively and stay constant up to 8 GHz and show a small fluctuation between 8 and 18 GHz. It is well known that the resonance behavior of the permittivity originates from the electron polarization, ion polarization, space charge polarization, dipole polarization, and interfacial polarization^34^. The ion polarization and electron polarization often take place in the range of THz and PHz^35^, the resonance of permittivity are believed to be from the space charge polarization, dipole polarization, and interfacial polarization. For Sample S700, a layer of covered dielectric α-Fe_2_O_3_ effectively decreases the electric conductivity and enhances the space charge polarization. Meanwhile, the dielectric α-Fe_2_O_3_ layer also introduces additional interfacial polarization charges^36^. In addition, with the increase of frequency, the dipole polarization was reported to be the dominant factor, resulting in the fluctuation of complex permittivity^37^, which is consistent with our experimental results (Figure 4b). For the existence of dielectric α-Fe_2_O_3_, it is reasonable to deduce that the dipole polarization of S700 is larger than that of S800. Based on the above discussion, the ε″ of S700 is higher than those of S800. The ε″ valley of S600 is supposed to be from the γ-Fe_2_O_3_ phase.

Figure 4c and 4d show the real part (μ′) and imaginary part (μ″) of the complex permeability of sample S600, S700 and S800. Except the range of 8–12 GHz, all of them show a similar frequency dependence of the μ′. The imaginary part μ″ is often used to indicate the magnetic loss. The μ″ of S600 decrease with the increasing frequency from 2 to 9 GHz and exhibit broad peak in the 9–14.4 GHz range due to the γ-Fe_2_O_3_ phase with high permeability, then keep almost unchanged in the 14.4–18 GHz with a minor fluctuation. The broad peak in the 9–14.4 GHz range can be attributed to the existence of γ-Fe_2_O_3_ phase. The slightly lower μ″ value of S700 than S800 in the range of 2–16 GHz means a smaller magnetic loss. In general, magnetic loss is reported to be dependent of magnetic hysteresis, domain-wall resonance, eddy current effect, exchange resonance and natural resonance. Considering the weakly applied magnetization field during the electromagnetic measurement^38^, magnetic hysteresis could be neglected. The domain-wall resonance is reported to takes place in the MHz range, and can also be excluded^39^. For S700, the eddy current effect is effectively suppressed and can also be excluded due to the existence of the insulative α-Fe_2_O_3_ layer. Therefore, the magnetic loss of S700 is mainly from the exchange resonance and natural resonance. Based on the reported exchange resonance frequency at about 12 GHz for cobalt^36^, the peak of μ″ around 17 GHz can be attributed to the exchange resonance based on the Aharoni theory^40^.

Figure 4e and 4f show the dielectric and magnetic tangent loss for the three samples, respectively. For S600, in the range of 10–15 GHz, the tanδ_E_ is low; while in other frequency range, it is high. On the other hand, the tanδ_M_ shows an opposite trend to that of tanδ_E_. These results suggest that in the low frequency range, the microwave absorption mainly results from the dielectric loss of α-Fe_2_O_3_, while in the high frequency range, magnetic loss of γ-Fe_2_O_3_ dominates. For both S700 and S800, tanδ_E_ values are relatively low in the low frequency range (2–10 GHz) and then increase in the range of 10–18 GHz with a fluctuation, while the tanδ_M_ of both samples are high in the low frequency (2–10 GHz) range and then decrease with the increasing frequency. Noteworthily, in the range of 16–18 GHz, S700 shows an abrupt increase, similar to the μ″. Although the magnetic loss of S700 is lower than that of S800, the effective complementarity of the complex permittivity and permeability is more important to improve the microwave absorption property than merely a high magnetic loss, which can explain the microwave absorption of S700 is superior to S800.

**Figure 5:**
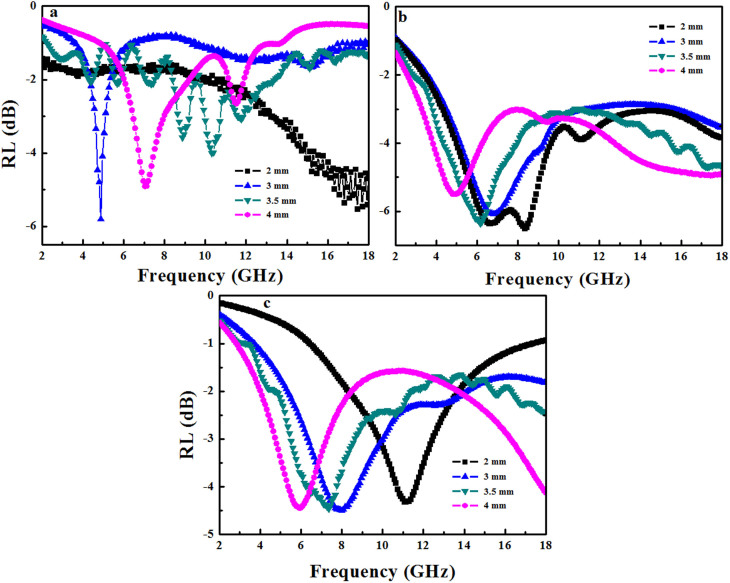
Electromagnetic wave RL of (a) mixture of Fe_3_O_4_ and rGO, (b) commercial [Fe, Ni], (c) [Fe, Ni] after annealling at 800°C for 1 hour in Ar.

Moreover, the EM wave absorption performance of the mixture of separately prepared Fe_3_O_4_ powders and rGO sheets were studied. The Fe_3_O_4_ powders was fabricated by one-pot co-precipitation method based on ref 41. The XRD pattern, Raman spectrum and SEM images of Fe_3_O_4_ powders and Fe_3_O_4_ powders/rGO can be found in Supplementary Figure S10 and S11. Figure 5a shows the RL of Fe_3_O_4_/rGO mixture. Compared with our samples, the RL loss is only −6 dB at 3 mm thickness. This clearly demonstrates the importance of synthetic procedure in the control of the EM wave absorption properties.

Moreover, commercial absorber: [Fe,Ni] (Beijing Reintech Electronics Technologies Co. Ltd, China) was selected for the comparative study in the same frequency range. Figure 5b and c shows the RL of [Fe,Ni] before and after heating. The RL of the as-obtained [Fe,Ni] is only −7 dB at 3.5 mm thickness. After being heated at 800°C for 1 hour in Ar, the RL was decreased to −5 dB, both of which are much inferior to our samples. In contrast, the RL for sample S700 under the same conditions is −17 dB at 3.5 mm thickness. These results clearly demonstrate the advantages of the synthetic strategy, the great EM wave absorption properties of the as-prepared samples, and the excellent thermal stability of these samples.

In summary, we reported the synthesis of iron oxides/rGO composites via a facile thermochemical process using graphite oxide and FeSO_4_·7H_2_O. Through the regulation of the preparation temperature, we can obtain α-Fe_2_O_3_/rGO, γ-Fe_2_O_3_/rGO, and Fe_3_O_4_/rGO composites conveniently. Interestingly, graphite oxide and rGO sheets were shown to regulate the phase transition from α-Fe_2_O_3_ to Fe_3_O_4_ via γ-Fe_2_O_3_. The hydroxyl groups in graphene oxide sheets and the H_2_ gas generated from the thermal reduction of graphene oxide were believed to be responsible for the transformation from α-Fe_2_O_3_ to γ-Fe_2_O_3_ in the 540°C–700°C. The reducing gases (CO, CH_4_ and H_2_) may facilitate the transformation from γ-Fe_2_O_3_ to Fe_3_O_4_. Electromagnetic wave absorption studies indicated that these samples showed great EM wave absorption performances. With the increase of GO dosage, the absorption performances can be greatly improved. These samples perform much better than the mixture of rGO with Fe_3_O_4_, as well as commercial absorbers. Most importantly, our samples possess advantages for high temperature applications due to its excellent stability in high temperature (up to 800°C).

## Methods

### Synthesis of iron oxides/graphene composites

In a typical synthesis: 5.04 g (0.018 mol) FeSO_4_·7H_2_O was dissolved into 20 mL distilled water with stirring for 30 min. Graphene oxide was synthesized by a modified Hummers method^42^. The resulted concentration of GO aqueous solution was 4.0 mg mL^−1^.

The preparation of the iron oxides/rGO composites was based on ref 43. 63 mL GO aqueous solution was mixed with 20 mL FeSO_4_·7H_2_O aqueous solution with ultrasonication for 30 min and vigorous stirring for 1 h. Then, the mixed suspension was dried at 90°C for 24 h, and the resulted black solid bulk was put in a quartz boat and placed in the middle part of a quartz tube, which was mounted horizontally inside a furnace. A protective gas of high purity argon (99.999%) was passed through the quartz tube at a rate of 500 standard cubic centimeters per minute (sccm) for 10 min to purge the air in the tube. The system was then heated to expected temperatures at a heating rate of 3°C per minute and held at that temperature for 1 hour before it cooled down to room temperature in the protective gas. The exhaust was imported into high concentration of NaOH solution in order to absorb SO_2_ and SO_3_ gases produced from the reaction process. The control samples were prepared by directly heating FeSO_4_·7H_2_O powders in furnace with the same procedure without GO.

**Figure 6:**
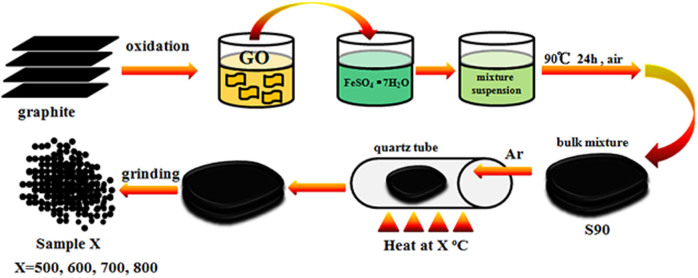
The schematic diagram of sample preparation process.

Figure 6 shows the schematic diagram of sample preparation process. Graphene oxide is negatively charged due to the rich electronegative oxygen species (epoxy, carboxyl, carbonyl and hydroxyl) on the surface and edges^44^. FeSO_4_·7H_2_O is easily dissolved in water and ionized into Fe^2+^ and SO_4_^2−^ ions. After mixing, the positively charged Fe^2+^ will be adsorbed on the GO sheets due to the electrostatic interaction, leading to the intercalation of Fe^2+^ and SO_4_^2−^ ions into GO layers^45^. The subsequent high temperature treatment could reduce graphene oxide into rGO and oxidize FeSO_4_·7H_2_O to Fe_2_O_3_^24^,^46^, forming Fe_2_O_3_/rGO composite.

### Electromagnetic wave absorption tests

The samples used for microwave absorption test were prepared by homogeneously mixing the composites with paraffin and pressed into a toroid with an outer diameter of 7.0 mm and inner diameter of 3.04 mm. The relative complex permittivity and permeability of the paraffin composites containing 50 wt% of the samples were determined by using a coaxial method with the vector network analyzer (Agilent E5071C, Agilent, USA) in the frequency range of 2–18 GHz. Finally, the microwave absorption properties were evaluated by the transmission line theory.

### Characterization

The structures, microstructures and morphologies of the as-obtained samples were characterized by X-ray powder diffraction (XRD Bruker D8-ADVANCE) using an 18 kW advanced X-ray diffractometer with Cu Kα radiation (λ = 1.54056 Å), Raman spectroscopy (inVia-Reflex, Renishaw, UK), Field-emission scanning electron microscopy (FE-SEM, S4800, Hitachi, Japan), Transmission electron microscopy (TEM, JEM-2100, JEOL, Japan), Fourier transform infrared microscopy (VERTEX 80+HYPERION2000, Bruker Optics, Germany), Simultaneous thermal analyzer ((DSC/DTA-T))STA 449 F3 Jupiter®, Netzsch, Germany). Magnetic properties were measured by Physical Property Measurement System (PPMS-9T (EC-II), Quantum Design, USA). Barrett-Emmett-Tellter (BET) method in the relative pressure P/P_0_ range of 0.01–0.20 was applied for the calculation of the pore volume, which was determined from the adsorption branch of the N_2_ isotherm curve at the P/P_0_ = 0.97 signal point. The pore diameter was derived from the maximum of the pore size distribution curve obtained using Barrett-Joyner-Halenda (BJH) method based on the adsorption branch of the N_2_ isotherm curve.

## Author Contributions

L.Z., X.Y. and H.H. designed and carried out the project. Z.L., Y.L., M.W. and C.C. wrote the manuscript. Z.W. and G.L. measured the electromagnetic wave absorption. Z.S. characterized the samples. All authors contributed to discussions of the results. All authors reviewed the manuscript.

## Supplementary Material

Supplementary Information Supplementary Information

## Figures and Tables

**Table 1:** The values of ε′, ε″, μ′, μ″, tanδ_E_ and tanδ_M_ of S600, S700 and S800

Samples	ε′	ε″	μ′	μ″	tanδ_E_	tanδ_M_
S600	16.88–24.44	1.21–7.61	0.89–1.32	0.05–0.35	0.28–0.43	0.05–0.31
S700	7.74–11.16	1.44–4.08	0.93–1.26	−0.22–0.23	0.13–0.50	−0.21–0.20
S800	6.92–8.18	0.44–2.35	0.86–1.36	−0.17–0.36	0.06–0.33	−0.19–0.30
