# Supplementary material for: Facile synthesis of iron oxides/reduced graphene oxide composites: application for electromagnetic wave absorption at high temperature
Source: Sci Rep. 2015 Mar 19;5:9298. doi: 10.1038/srep09298 (PMC4365402; doi:10.1038/srep09298)
Supplement: Supplementary Information [file srep09298-s1.doc]

**Supplementary Information**

**Facile synthesis of iron oxides/reduced graphene oxide composites: application for electromagnetic wave absorption at high temperature**

Lili Zhang^1^, Xinxin Yu^1^, Hongrui Hu^1^, Yang Li^1^, Mingzai Wu^1*^, Zhongzhu Wang^1^, Guang Li^1^, Zhaoqi Sun^1^ & Changle Chen^2*^

^1^School of Physics and Materials Science, Anhui University, Hefei 230601, China ^2^CAS Key Laboratory of Soft Matter Chemistry, Department of Polymer Science & Engineering, University of Science and Technology of China, Hefei 230026, China

*Author to whom correspondence should be addressed.

E-mail: [mingzaiwu@gmail.com](mailto:mingzaiwu@gmail.com), [changle@ustc.edu.cn](mailto:changle@ustc.edu.cn) Tel: 86-551-63861813;

Fax: 86-551-63861813.

**Supplementary Figure legends**

Supplementary Figure S1 XRD patterns of FeSO_4_•7H_2_O powders, S540, S_0_600, S_0_700, S_0_800.

Supplementary Figure S2 Raman spectral of S500, S600, S700 and S800. (laser power: 0.5mW; integration time: 30 seconds).

Supplementary Figure S3 FE-SEM images of (a, b) S600, (c, d) S700, (e, f) S800.

Supplementary Figure S4 FT-IR spectral of GO, S90, S500, S600, S700, S800.

Supplementary Figure S5 TG (a) and DTG (b) curves of GO, FeSO4•7H_2_O, S90.

Supplementary Figure S6 (a) N_2_ adsorption-desorption isotherm, (b) pore size distribution plots of S800.

Supplementary Figure S7 The photographs of (a) S90, (b) S600, (c) divided S600, (d) grounded S600 powders, (e, f) magnetic attraction.

Supplementary Figure S8 The photographs of (a) S90, (b) S700, (c) divided S700, (d) grounded S700, (e, f) magnetic attraction.

Supplementary Figure S9 (a) Schematic diagram of the transformation of γ-Fe_2_O_3_ into Fe_3_O_4_, (b) The percentage ratio of α-Fe_2_O_3_ phase on the surface to the inner Fe_3_O_4_ phase was enhanced by grounding S90, (c) XRD pattern of ground sample annealed at 800^o^C for 1h, (d) Magnetic hysteresis loop of S800. (Inset a) ground S90 heated at 800 oC, (inset b) the enlarged low field hysteresis loop of S800.

Supplementary Figure S10 XRD patterns of Fe_3_O_4_ powders, commercial electromagnetic wave absorber: [Fe, Ni] and [Fe, Ni] after heating at 800 ^o^C in Ar for 1 hour. The inset is the Raman spectrum of Fe_3_O_4_ powders.

Supplementary Figure S11 SEM images of (a) as-obtained Fe_3_O_4_, (b) mixture of Fe_3_O_4_ powders/rGO sheets, (c) commercial [Fe, Ni], (d) [Fe, Ni] after annealing at 800 ^o^C for1 hour in argon.

Supplementary Figure S1 shows XRD patterns of FeSO_4_•7H_2_O, S540, S_0_600, S_0_700 and S_0_800. The patterns marked with “●” for S_0_ are well indexed to FeSO_4_•7H_2_O. After being annealed at 600 ^o^C, FeSO_4_•7H_2_O was oxidized into Fe_2_(SO_4_)_3_ and partial Fe_2_(SO_4_)_3_ decomposed into α-Fe_2_O_3_. With the increase of annealing temperature to 700 ^o^C and 800 ^o^C, all of the peaks for S_0_700 and S_0_800 can be well indexed to α-Fe_2_O_3_. As for S540, all the peaks belong to α-Fe_2_O_3_. Both XRD data of S540 and S_0_600 indicate that the introduction of GO sheets in S540 decreased the thermal stability of FeSO_4_•7H_2_O or the intermediate products.

**
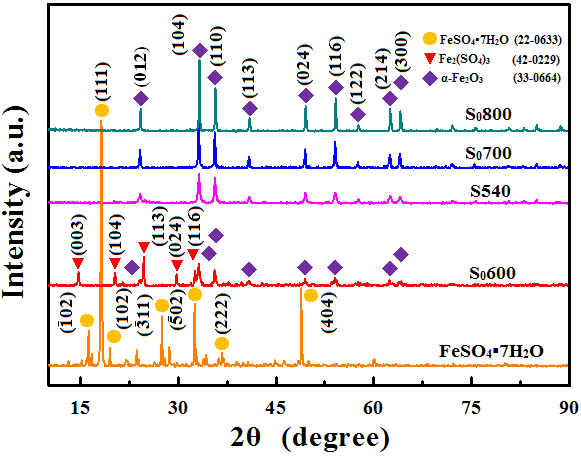
**

Supplementary Figure S1

Micro-Raman analysis with low laser power and relative long integration time was performed to avoid any phase transition. Supplementary Figure S2 shows Raman spectral of S500, S600, S700 and S800. Two characteristic peaks at ~1346cm^-1^ and ~1605 cm^-1^ are observed for all samples, which correspond to “D” and “G” bands from the defects and disorders caused by sp^3^-carbon atoms and the plane vibration of the sp^2^-carbon atoms in two-dimensional lattice of rGO^1^. For S500, three peaks at 225cm^-1^, 294cm^-1^ and 412cm^-1^ are ascribed to A_1g_ and two E_g_ vibrational modes of α-Fe_2_O_3_^2, 3^. For S600, two new peaks at 500 cm^-1^ (E_g_ mode) and 700cm^-1^ (A_1g_ mode) indicate the formation of γ-Fe_2_O_3_^2, 3^; While for S700 and S800, the characteristic peaks at 666cm^-1^ (A_1g_ mode) and 540cm^-1^ (T_1g_ mode) confirm the formation of Fe_3_O_4_ phase^2, 3^.

**
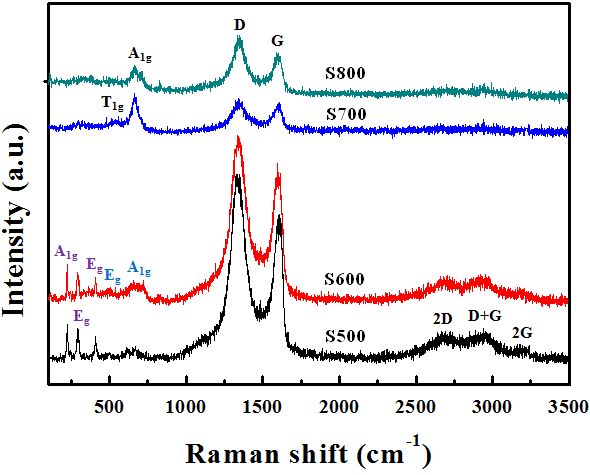
**

Supplementary Figure S2

Supplementary Figure S3 shows the FE-SEM images of S600, S700 and S800. Iron oxides particles with diameters of ca. 230 nm are embedded tightly on the surface of rGO sheets, with some particles distributed in the space between rGO layers. Curly crumples are clearly observed, which is from rGO. The interspaces formed in composites (Supplementary Figure S3f) play a key role in the phase transition from γ-Fe_2_O_3_ to Fe_3_O_4_, which will be discussed in the main manuscript.

**
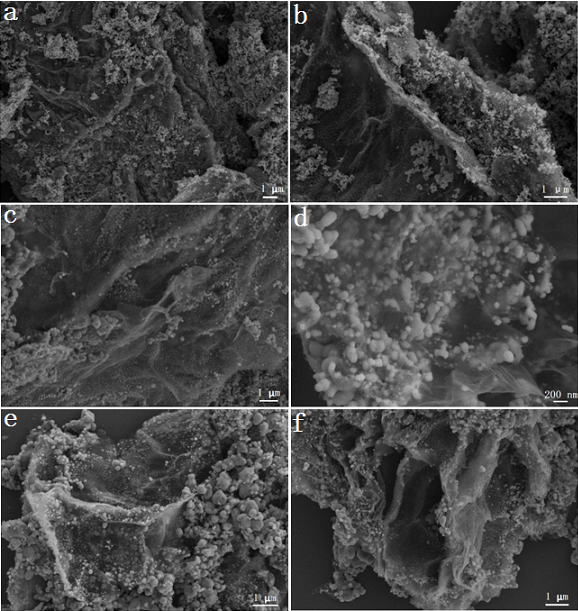
**

Supplementary Figure S3

Supplementary Figure S4 shows the FT-IR spectral of GO and five samples. For GO, the characteristic peaks of various carbon-oxygen functional groups are detected, which agree with references^4, 5^. The peaks of GO are preserved for S90. In addition, the peaks at 530 cm^-1^ and 624 cm^-1^ correspond to ν_4_ bending mode of the SO_4_^2-^ units^6^ and the peak at 824cm^-1^ is assigned to the stretching vibration of S-O bonds. Peaks at 998cm^-1^, 1017cm^-1^ and 1083cm^-1^, 1147cm^-1^ are assigned to symmetric stretching vibration of the SO_4_^2-^ units^6^ and ν_3_ anti-symmetric stretching of SO_4_^2,7^. The peak at 665cm^-1^ is attributed to the rocking vibration of water molecules in FeSO_4_•H_2_O. All of the peaks confirm the existence of FeSO_4_•H_2_O in the composite, which agrees well with the XRD analysis of S90. For S500, the disappearance of peaks at 624cm^-1^ and 824cm^-1^ and the intensity decrease of peaks at 997cm^-1^, 1070cm^-1^ and 1140cm^-1^ are attributed to partial decomposition of FeSO_4_•H_2_O to α-Fe_2_O_3_, H_2_O, SO_2_ and O_2_^8^. New peaks at 448cm^-1^, 474cm^-1^ and 539cm^-1^ indicate the formation of α-Fe_2_O_3_^3^. The decrease of the peaks at 880cm^-1^ and 3430cm^-1^ indicate the partial removal of epoxide and –COOH groups from graphite oxide sheets^4, 9^. The intensity decrease of the peak at 1630cm^-1^ and disappearance of peaks at 1385cm^-1^ and 2976cm^-1^ can be attributed to the vaporization of intercalated water and dehydration of FeSO_4_•H_2_O. The absorption peaks at 1584cm^-1^, 1630cm^-1^ and 997cm^-1^ can be assigned to C=C skeletal vibrations of rGO^4, 5^. The symmetric stretching vibration of SO_4_^2-^ ions suggests the existence of FeSO_4_. As for sample S600, all of the peaks from SO_4_^2-^ ions disappear. The existence of peaks at 880cm^-1^ and 3430cm^-1^ indicates the residual epoxide and hydroxyl groups in the sample. The two peaks at 470cm^-1^ and 551cm^-1^ can be ascribed to α-Fe_2_O_3_, and the two peaks at 580cm^-1^ and 620cm^-1^ indicate the presence of γ-Fe_2_O_3_^10^. Most of characteristic peaks from GO disappeared in the spectral of S700 and S800, indicating the complete removal of epoxide and hydroxyl groups. The peaks at 448cm^-1^ and 577cm^-1^ correspond to the vibrational modes of α-Fe_2_O_3_ and Fe_3_O_4_^3, 10^. For S800, only peaks of Fe_3_O_4_ are detected. These results are fully consistent with the XRD and Raman analysis.


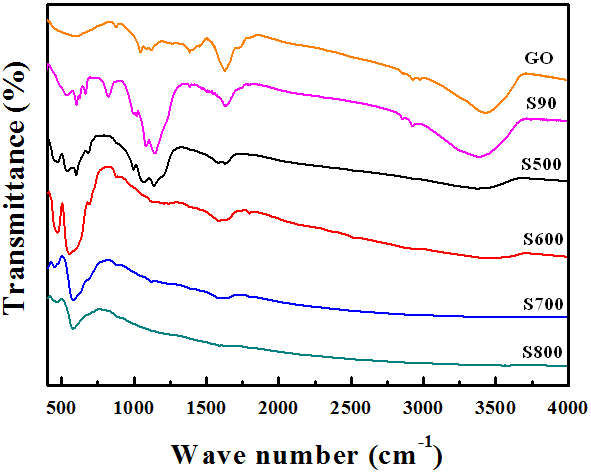


Supplementary Figure S4

TG/DTG measurements were carried out for GO, FeSO_4_•7H_2_O and S90 in nitrogen atmosphere with a heating rate of 10 ^o^C/min (Supplementary Figure S5). Four obvious reactions took place in the process: (I) mild vaporization of intercalated water molecules from 45 ^o^C to 140 ^o^C; (II) drastic vaporization of intercalated water molecules from 140 ^o^C to 220 ^o^C with the maximum weight loss at 180 ^o^C; (III) removal of most carboxyl groups (-COOH) between 220 ^o^C and 600 ^o^C along with the formation of CO_2_, CO, CH_4_ and H_2_ , which agrees well with references^11, 12^. FT-IR analysis shows the complete removal of residual epxoide and hydroxyl groups for S600, S700 and S800, which suggests another thermochemical reaction: (IV) removal of residual epoxide and hydroxyl groups from 600 ^o^C to 800 ^o^C^11, 12^, which is supported by 5% weight loss in the TG curve.

For FeSO_4_•7H_2_O, 9%, 28% and 35% weight are lost at 58 ^o^C, 140 ^o^C and 270 ^o^C, which agrees with the theoretical value of thermal dehydration of ferrous sulphate tetrahydrate (FeSO_4_•4H_2_O)^8^. FeSO_4_•7H_2_O is stable up to 42 ^o^C^13^ and three water molecules would be lost when it was placed in our TG/DTG test system (starting test temperature is 45 ^o^C). At about 270 ^o^C, FeSO_4_•4H_2_O lose the entire water molecule, forming FeSO_4_, which decomposed at about 510 ^o^C. The whole decomposition process was completed from 510 ^o^C to 650 ^o^C with the maximum weight loss temperature at 578 ^o^C. These results are consistent with previous report^8, 13-15^. In addition, there are two apparent endothermic peaks positioned at 578 ^o^C and 621 ^o^C, as shown in Supplementary Figure S5, which indicates that the decomposition process of FeSO_4_ into Fe_2_(SO_4_)_3_ and α-Fe_2_O_3_ is step by step^8, 13, 14^.

The TG and DTG curves of S90 can be divided into three stages: (I) vaporization of intercalated water molecules from GO between 70 ^o^C and 210 ^o^C; (II) removal of -COOH groups of GO and dehydration of water molecule from FeSO_4_•H_2_O between 230 ^o^C and 330 ^o^C; (III) decomposition of FeSO_4_ into α-Fe_2_O_3_ between 430 ^o^C and 580 ^o^C with the maximum weight loss at 538 ^o^C. Compared with the curves of pure FeSO_4_•7H_2_O, the starting decomposition temperature and the maximum weight loss temperature of S90 are shifted to lower temperature by about 80 ^o^C (510 ^o^C shifted to 430 ^o^C) and 40 ^o^C (578 ^o^C shifted to 538 ^o^C), respectively. The weight loss is not obvious in the range of 600 ^o^C to 800 ^o^C, which is consistent with the slight weight differences among α-Fe_2_O_3_, γ-Fe_2_O_3_ and Fe_3_O_4_. From DTG curves of S90, the endothermic peak at 538 ^o^C can be identified as the decomposition temperature of FeSO_4_ to α-Fe_2_O_3_^8, 13, 14^, which is supported by the XRD patterns of S540 (Supplementary Figure S1). In addition, to better understand the mechanism of phase transition of iron oxides, the summary of the thermal reactions of graphite oxide, FeSO_4_•7H_2_O and S90 in the 45 ^o^C - 800 ^o^C range are listed in Supplementary Table S1.


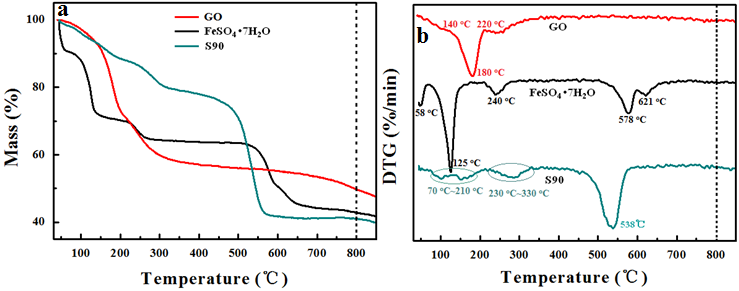


Supplementary Figure S5

Supplementary Table S1 The summary of thermochemical rections of graphite oxide, FeSO_4_•7H_2_O and S90

| Temp.(^o^C) | graphite oxide | FeSO_4_•7H_2_O | S90 |
| --- | --- | --- | --- |
| 45 |  | dehydrates 3 water molecules to form FeSO_4_•4H_2_O |  |
| 90 |  |  | dehydrates 6 water molecules to form FeSO_4_•H_2_O and mild vaporization of intercalated water in air during longtime drying of GO |
| 45 - 140 | mild vaporization of intercalated water |  |  |
| 80 - 150 |  | FeSO_4_•4H_2_O dehydrates 3 water molecules to form FeSO_4_•H_2_O |  |
| 70 - 210 |  |  | GO: mild and drastic vaporization of intercalated water |
| 140 - 220 | drastic vaporization of intercalated water |  |  |
| 210 - 270 |  | FeSO_4_•H_2_O dehydrates last one water molecule to form FeSO_4_ |  |
| 220 - 600 | removal of most reliable carboxyl groups | FeSO_4_ decomposes into α-Fe_2_O_3_, SO_2_ and O_2_ (510 ^o^C-650 ^o^C) | decomposition of FeSO_4_ to form α-Fe_2_O_3_, SO_2_ and O_2_ (460 ^o^C -580 ^o^C) |
| 600 - 800 | removal of residual carboxyl and hydroxyl groups |  | α-Fe_2_O_3_ transforms into γ-Fe_2_O_3_ (540 ^o^C -700 ^o^C),  γ-Fe_2_O_3_ transforms into Fe_3_O_4_ (600 ^o^C -800 ^o^C) |

**
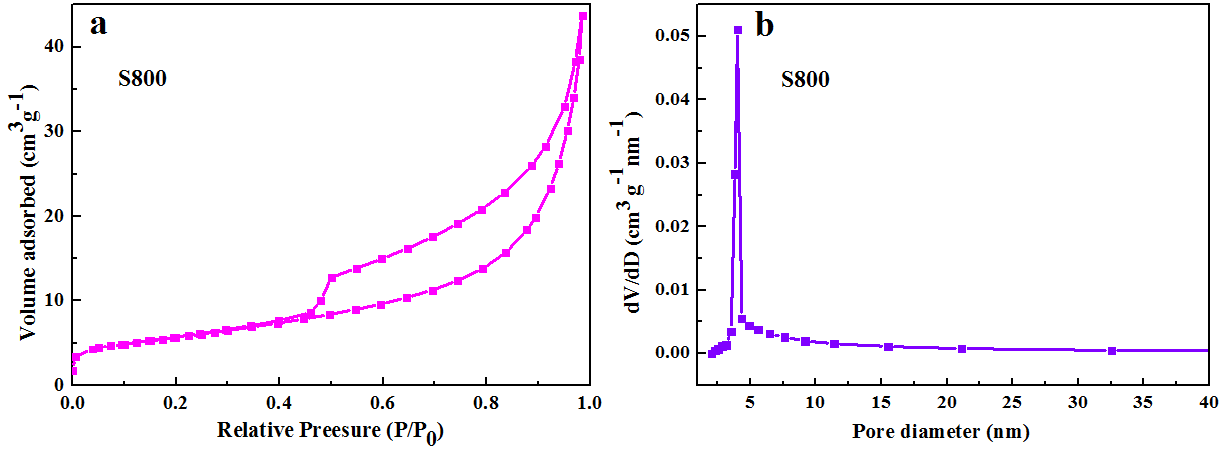
**

Supplementary Figure S6


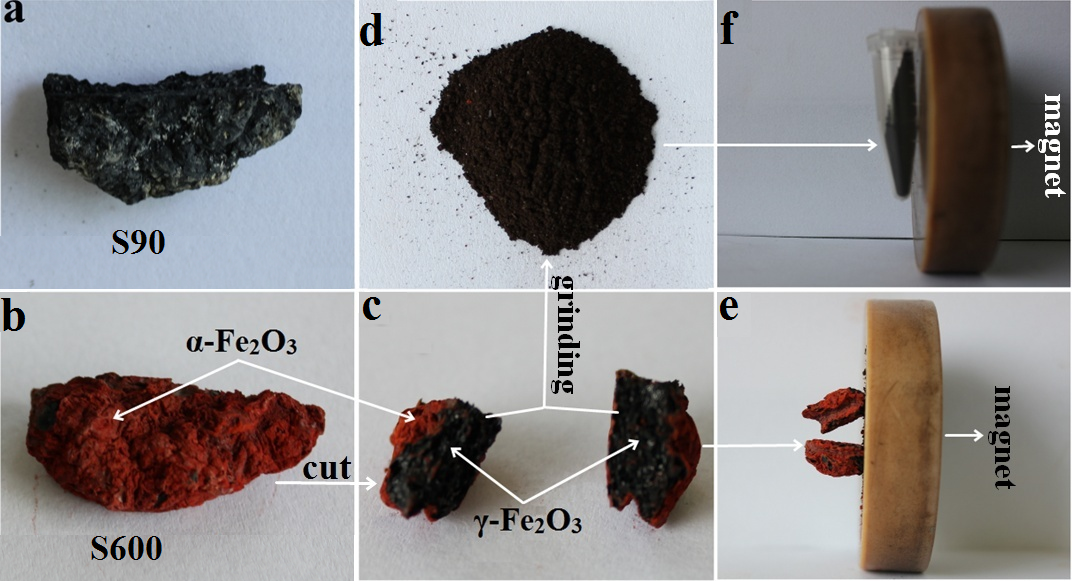


Supplementary Figure S7


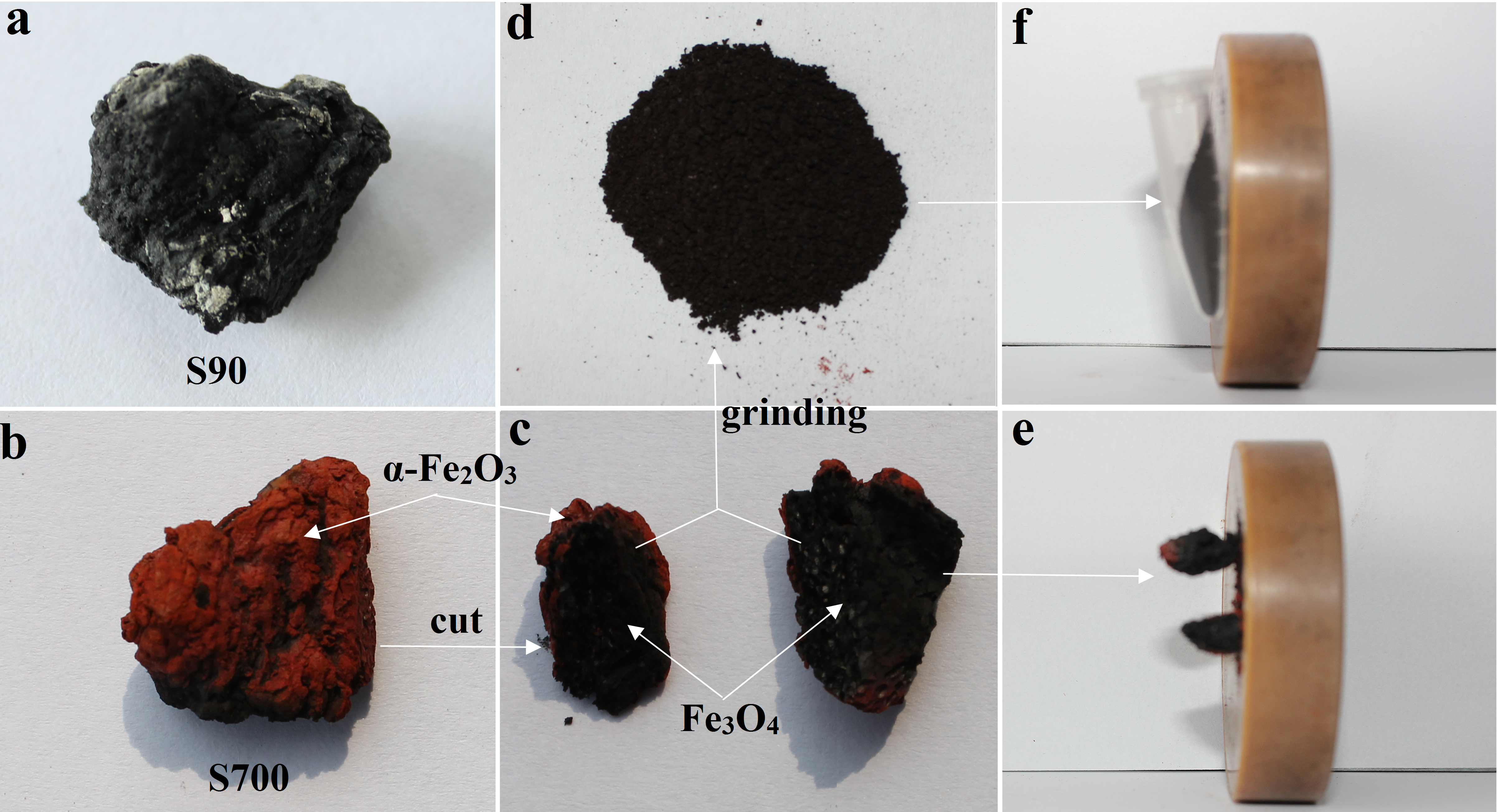


Supplementary Figure S8


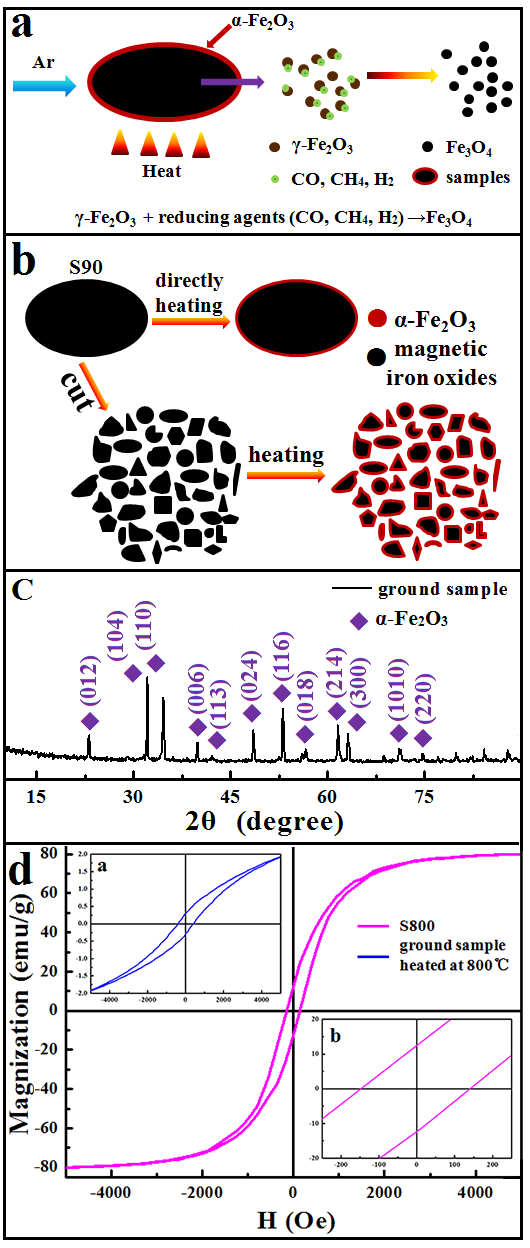


Supplementary Figure S9


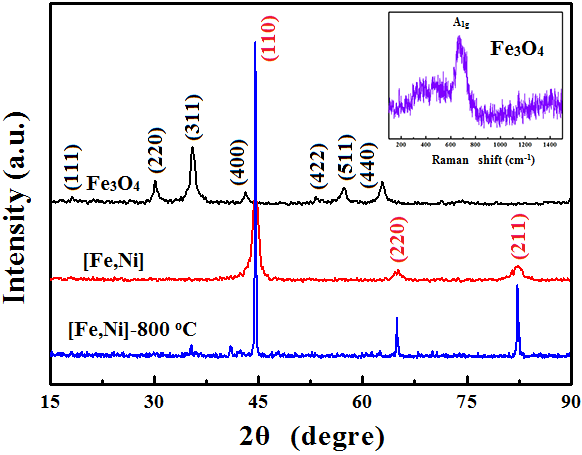


Supplementary Figure S10


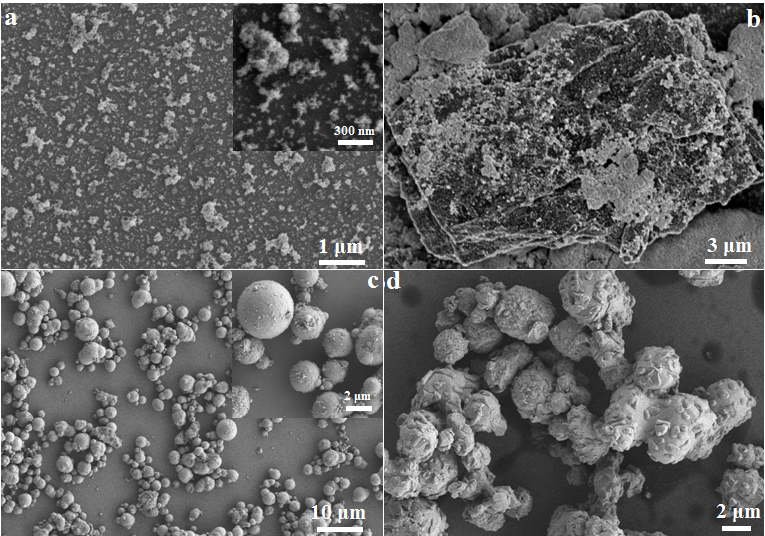


Supplementary Figure S11

**Reference**

1. Malard, L., Pimenta, M., Dresselhaus, G. & Dresselhaus, M. Raman spectroscopy in graphene. *Phys. Rep.* **473**, 51-87 (2009).

2. De Faria, D., Venâncio Silva, S. & De Oliveira, M. Raman microspectroscopy of some iron oxides and oxyhydroxides. *J. Raman Spectrosc.* **28**, 873-878 (1997).

3. Jubb, A. M. & Allen, H. C. Vibrational spectroscopic characterization of hematite, maghemite, and magnetite thin films produced by vapor deposition. *ACS Appl. Mater. Interfaces* **2**, 2804-2812 (2010).

4. Lin, Z.et al. Solvent-assisted thermal reduction of graphite oxide. *J. Phys. Chem. C* **114**, 14819-14825 (2010).

5. Acik, M. et al. The role of oxygen during thermal reduction of graphene oxide studied by infrared absorption spectroscopy. *J. Phys. Chem. C* **115**, 19761-19781 (2010).

6. Frost, R.L., Wills, R.-A., Kloprogge, J. T. & Martens, W. N. Thermal decomposition of hydronium jarosite (H_3_O)Fe_3_(SO_4_)_2_(OH)_6_. *J. Therm. Anal. Cal.* **83**, 213-218 (2006).

7. Zhou, J.et al. Infrared spectroscopy of hydrated sulfate dianions. *J. Chem. Phys.* **125**, 111102 (2006).

8. Masset, P., Poinso, J.-Y. & Poignet, J.-C. TG/DTA/MS study of the thermal decomposition of FeSO_4_·6H_2_O. *J. Therm. Anal. Cal.* **83**, 457-462 (2006).

9. Kaniyoor, A., Baby, T. T. & Ramaprabhu, S. Graphene synthesis via hydrogen induced low temperature exfoliation of graphite oxide. *J. Mater. Chem.* **20**, 8467-8469 (2010).

10. Cornell, R. M., & Schwertmann, U. The Iron Oxides: Structure, Properties, Reactions, Occurrences and Uses [Cornell, R. M., & Schwertmann, U. (ed.)] [139-184] (WILEY-VCH Verlag GmbH & Co. KGaA, Weinheim, 2003).

11. Huh, S. H. [Thermal reduction of graphene oxide] Physics and Applications of Graphene-Experiments [Mikhailov, S (ed.)] [73-90] (InTech, Shanghai, 2011).

12. Jung, I. et al. Reduction kinetics of graphene oxide determined by electrical transport measurements and temperature programmed desorption. *J. Phys. Chem. C* **113**, 18480-18486 (2009).

13. Swami, M., Prasad, T. & Sant, B. Thermal analysis of ferrous sulphate heptahydrate in air. II. The oxidation-decomposition path. *J. Therm. Anal.* **16**, 471-478 (1979).

14. Petkova, V. & Pelovski, Y. Comparative DSC study on thermal decomposition of iron sulphates. *J. Therm. Anal. Cal.* **93**, 847-852 (2008).

15. Gallagher, P., Johnson, D. & Schrey, F. Thermal decomposition of iron (II) sulfates. *J. Am. Ceram. Soc.* **53** , 666-670 (1970).
